# Antimicrobial Resistance of Seventy Lactic Acid Bacteria Isolated from Commercial Probiotics in Korea

**DOI:** 10.4014/jmb.2210.10041

**Published:** 2023-01-12

**Authors:** Eunju Shin, Jennifer Jaemin Paek, Yeonhee Lee

**Affiliations:** 1 Culture Collection of Antimicrobial Resistant Microbes, Department of Horticulture, Biotechnology, and Landscape Architecture, Seoul Women’s University, Seoul 01797, Republic of Korea; 2 PLBNB, Guri 11960, Republic of Korea

**Keywords:** Antimicrobial susceptibility, antimicrobial resistance gene, MDR, lactic acid bacteria, probiotics, safety

## Abstract

In this study, lactic acid bacteria were isolated from 21 top-selling probiotic products on Korean market and their antimicrobial resistance were analyzed. A total 152 strains were claimed to be contained in these products and 70 isolates belonging to three genera (*Bifidobacterium*, *Lactobacillus*, and *Lactococcus*) were obtained from these products. RAPD-PCR showed diversity among isolates of the same species except for two isolates of *Lacticaibacillus rhamnosus* from two different products. The agar dilution method and the broth dilution method produced different MICs for several antimicrobials. With the agar dilution method, five isolates (three isolates of *Bifidobacterium animalis* subsp. *lactis*, one isolate of *B. breve*, one isolate of *B. longum*) were susceptible to all nine antimicrobials and 15 isolates were multi-drug resistant. With the broth microdilution method, only two isolates (one isolate of *B. breve* and one isolate of *B. longum*) were susceptible while 16 isolates were multi-drug resistant. In this study, only two AMR genes were detected: 1) *lnu*(A) in one isolate of clindamycin-susceptible and lincomycin-resistant *Limosilactobacillus reuteri*; and 2) *tet*(W) in one tetracycline-susceptible isolate of *B. longum* B1-1 and two tetracycline-susceptible isolates and three tetracycline resistant isolates of *B. animalis* subsp. *lactis*. Transfer of these two genes via conjugation with a filter mating technique was not observed. These results suggest a need to monitor antimicrobial resistance in newly registered probiotics as well as probiotics with a long history of use.

## Introduction

Lactic acid bacteria (LABs) have been widely used as probiotics and starter cultures worldwide. Due to a long history of safe consumption, many LAB species are considered as generally recognized as safe (GRAS) according to the FDA (US Food and Drug Administration) with a qualified presumption of safety (QPS) status provided by EFSA (European Food Safety Authority) [1-3]. Although LABs are considered as safe in general, worldwide concern about antimicrobial resistance (AMR) has been increasing. AMR of LABs has been suspected as a reservoir for antimicrobial resistance to human intestinal microbiota by transferring resistance [4-9]. Since LABs are generally consumed more than 10^8^ CFU/day, extrinsic resistance in LAB can be transferred to normal microbiome, rendering a major health problem [10]. Many countries have started to distinguish between intrinsic and acquired resistance of LABs and regulate the use of LABs with acquired resistance as a probiotic [11]. EFSA guidance for safety assessment of acquired AMR genes in probiotics, starter cultures, or feed additives in the EU [12] and defined microbiological cut-off values (MCOFFs) of certain antimicrobials to identify strains carrying acquired AMR genes. Phenotypic antimicrobial susceptibility is determined by defining the minimum inhibitory concentration (MIC) values for antimicrobials listed in the guidance [12]. Strains with higher MIC values than the defined cut-off values may carry acquired AMR genes and require more investigation.

In Korea, a greatly diverse commercial probiotics with various formulations are on the market with market size being expanding significantly every year. Many probiotic strains on the market in Korea have been imported from other countries. As the demand for safe LABs has been increasing, antimicrobial susceptibility testing for a newly registered LAB strain has been mandatory in Korea since 2021. However, the ones already on the market are exempt from this guideline.

This study was conducted to assess the safety of LABs in probiotic products of Korea, focusing on antimicrobial resistance. To accomplish this, antimicrobial susceptibility testing, detection of AMR genes with PCR, and transfer of AMR genes via conjugation were performed. In addition, random amplified polymorphic DNA (RAPD)-PCR was performed to compare isolates belonging to the same species contained in different products.

## Materials and Methods

### Samples

A total 21 top-selling probiotic products in Korea were purchased from the market. Nine products were selected based on “Trend Analysis of Health Functional Food in 2016” (https://www.mfds.go.kr/search/search.do) by Korea Food and Drug Administration. The rest 13 products were online top-selling products.

### Isolation of LABs from Commercial Probiotic Products

One gram of each sample was dispersed in nine ml of sterile saline and shaken for 1 h at room temperature. These samples were 10-fold serially diluted with sterile saline and then 0.1 ml of each diluted sample was inoculated and spread onto de Man Rogosa Sharpe (MRS, BBL Becton Dickinson, Sparks, MD, USA) agar with 0.005% bromophenol blue [13] for *Lactobacillus* (hereafter “*Lactobacillus*” refers to the all-new genera reclassified such as *Lacticaseibacillus* and *Lactiplantibacillus* unless mentioned otherwise) and modified *Bifidobacterium* agar [14] for *Bidifobacterium*, Trypticase soy agar (BBL) with 5% defibrinated sheep blood, Enterococcossel agar (BBL), and *Streptococcus thermophilus* isolation agar (Sigma-Aldrich, USA) were used to isolate strains belonging to genera *Bacillus*, *Enterococcus*, and *Streptococcus*, respectively. Plates were incubated in a Gaspak jar (BBL) at 37°C. After incubation at 37°C for 48 h, colonies with different shapes, sizes, and colors were selected and passaged three times to obtain pure colonies. Well isolated colonies presumed to be LAB were dispersed in 20% glycerol and stored in an ultra-low freezer (less than -70°C) for further studies.

### Genomic DNA Extraction

Bacterial cells were collected from well isolated colonies, suspended in sterile saline, and then harvested by centrifugation at 14,000 ×*g* for 10 min. Genomic DNA was eluted with 30 μl of 10 mM TE buffer (10 mM TrisHCl, 1 mM EDTA, pH 8.5) using a G-spin genomic extraction kit (Intron Biotechnology, Korea) and stored at −20°C.

### Identification with 16S rRNA Gene Sequencing

To identify species, PCR amplification of the 16S rRNA gene was performed with the following primers: 27F, 5¢-AGA GTT TGA TCM TGG CTC AG-3¢ and 1088R, 5¢-GCT CGT TGC GGG ACT TAA CC-3¢ [15]. PCR was performed in a GeneAmp 9700 thermocycler (Applied Biosystems, USA) with the following thermal cycling conditions: pre-denaturation at 95°C for 5 min, 30 cycles of 95°C for 30 sec, 57°C for 30 sec, and 72°C for 45 sec, followed by 10 min at 72°C. DNA fragments were purified using a QIAquick Gel Extraction Kit (Qiagen, USA) in accordance with the manufacturer’s instruction. Sequence reactions were performed with an ABI 3730XL DNA analyzer (Applied Biosystems) by Bionix (Korea). Sequences were analyzed using the BLAST algorithm at the National Center for Biotechnology Information web server (https://www.ncbi.nlm.nih.gov).

### Antimicrobial Susceptibility Testing

Minimal Inhibitory Concentrations (MICs) were determined with both agar dilution method and broth microdilution method in triplicates on different dates. LAB susceptibility test medium (LSM) consisting of a mixture of 90% Iso-Sensitest broth (IST, England) and 10% MRS broth adjusted to pH 6.7 was used for *Bifidobacterium* and *Lactobacillus* species and IST broth was used for *Lactococcus* following recommendations of the International Standard ISO 10932 [16]. The following 9 antimicrobials at various concentrations were tested as shown in Table 1: ampicillin, chloramphenicol, clindamycin, erythromycin, gentamicin, kanamycin, streptomycin, tetracycline and vancomycin. All chemicals were purchased from Sigma (USA) except chloramphenicol (Fluka, Switzerland). *Bifidobacterium* longum 15707, *L. paracasei* ATCC 334, *L. plantarum* ATCC 14917, and *Lc. lactis* subsp. *lactis* ATCC 19435 were included as controls in each batch of agar dilution method and broth microdilution tests [16]. Microdilution plates were incubated anaerobically in a Gaspak jar with a Gaspak anaerobic system for 48 h following the ISO 10932. Incubation temperatures were: 28°C for *L. brevis*, *L. plantarum*, and *L. sakei*; 32°C for *Lc. lactis* subsp. *lactis*: 37°C for other lactobacilli and bifidobacteria. Both agar dilution method and broth dilution method were performed in triplicates on different dates. Interpretation criteria for resistance to nine antimicrobials were defined as the MCOFFs by EFSA (2018).

### Detection of Antimicrobial Resistant Genes

PCR amplifications were performed with primers corresponding to 17 antimicrobial extrinsic resistant genes. Annealing temperature and resulting amplicon size are presented in Table 2 [17-26]. Reaction mixtures without DNA template were used as negative controls. Amplification products were analyzed by agarose gel electrophoresis and visualized in a gel documentation system (Bio-Rad). Resulting PCR products were sequenced at Bionix (Korea) and analyzed using the online BLAST algorithm at the National Center for Biotechnology Information web server (https://www.ncbi.nlm.nih.gov/).

### Conjugative Transfer with a Filter Mating Technique

Conjugation was performed in duplicates using a filter mating technique as described previously [24]. *E. faecalis* JH 2-2 (LMG 19456: fusidic acid^r^, rifampin^r^) and *Lc. lactis* subsp. *lactis* Bu2-60 (LMG 19460: streptomycin^r^, rifampin^r^) were used as recipients. A total of eight isolates consisting of two *Lactobacillus* isolates (tetracycline-resistant *Lacticaseibacillus rhamnosus* 2-7 without *lnu*(A); lincomycin- and tetracycline-resistant, and clindamycin-susceptible *Limosilactobacillus reuteri* 16-1 with *lnu*(A)) and six tetracycline-resistant *Bifidobacterium* isolates with *tet*(W) (*B. animalis* subsp. *lactis* B3-2, B8-1, B11, B 14-4, B 15-1, and *B. longum* B1-1) were selected as donors. *Lactobacillus*, *Lactococcus*, *Bifidobacterium*, and *Enterococcus* were grown in MRS broth, M17 (BBL) broth, MRS-cysteine (0.25%) broth, and Brain Heart Infusion (BBL) broth, respectively. One ml of the donor at the mid-exponential phase of growth was mixed with an equal volume of the recipient and the mixture was filtered through a sterile mixed cellulose esters filter (0.45 μm; MF-Millipore membrane filter, HAWP 02500, Millipore, USA) using a Swinnex filter holder (SX00 02500, Millipore). Peptone physiological saline solution (PPS, Oxoid, Basingstoke, Hants, UK) was passed through the filter to hold cells on the filter more tightly. The membrane was then placed on BHI agar for *Enterococcus* or MRS agar for *Lactococcus* without antimicrobial agents and incubated anaerobically at 37°C for 48 h. After incubation, the membrane was transferred into 2 ml PPS and cells were detached with shaking. The mated mixture was diluted and inoculated with spreading on BHI agar for *Enterococcus* or MRS agar for *Lactococcus* containing 10 μg/ml of tetracycline (Sigma) and 50 μg/ml of rifampicin (Sigma) and then incubated at 37°C for 48 h to select transconjugants. As controls, donor and recipient strains were individually plated onto appropriate agar plates (MRS agar for *Lactobacillus*, M17 agar for *Lactococcus*, MRS-cysteine agar for *Bifidobacterium*, and BHI agar for *Enterococcus*) containing 10 μg/ml of tetracycline (Sigma) and 50 μg/ml of rifampicin. Colonies grown on double selective media were inoculated on medium without antimicrobial and checked for coccid morphology under a microscope. PCR was performed with DNA extracted from transconjugants to detect *lnu*(A) or *tet*(W) gene.

### Random Amplified Polymorphic DNA (RAPD)-PCR

Random primers used for RAPD in this study are shown in Table 3 and synthesized by Bionix (Korea). The reaction was performed as described by Kern *et al*. [27] with some modifications. The reaction mixture (a final volume of 20 μl) contained 1× buffer, 2.5 mM MgCl_2_, 200 μM deoxynucleoside triphosphates, 2 μM primer, 1.25 U *Taq* polymerase (Intronbiotechnology, Korea), and 1 μl of genomic DNA prepared as described above. The amplification was performed in a GeneAmp 9700 thermocycler (Applied Biosystems, FUSA) with the following thermal cycling conditions: pre-denaturation at 94°C for 2 min, 40 cycles of 94°C for 15 sec, 35°C for 30 sec, and 72°C for 2 min, followed by 10 min at 72°C. After the reaction, 10 μl of each PCR product was analyzed on a 1%agarose gel. These gels were visualized with a gel documentation system (Bio-Rad, Italy).

## Results 

### Isolation and Identification of LABs from Commercial Probiotics

A total of 152 strains of bacteria were claimed to be contained in 21 top-selling probiotic products in Korea, including 131 strains belonging to genera *Bifidobacterium*, *Lacticaseibacillus*, *Lactiplantibacillus*, *Lactobacillus*, *Latilactobacillus*, *Levilactobacillus*, *Ligilactobacillus*, *Limosilactobacillus*, *Lactococcus* and 21 strains of *Bacillus coagulans* (3 strains), *B. subtilis* (1 strain), *Enterococcus faecalis* (2 strains), *E. faecium* (8 strains), and *Streptococcus thermophilus* (7 strains). Only 32 strains had strain numbers. When pure colonies were isolated from each product and identified, 70 (54%) of 131 LAB strains labelled on the products could be isolated and identified as presented in Table 4. These 70 LAB isolates belonged to 9 genera and 17 species while *B. bifidum* and *L. delbrueckii* subsp. *lactis* claimed on the labels of products were not recovered.

### Antimicrobial Susceptibility Tsting

Table 5 shows MICs to 9 antimicrobials of 70 LAB isolates from commercial probiotic products. MICs higher than MCOFFs were written in boldface and MICs 4 times higher than MCOFFs were underlined. Agar dilution method showed that 65 isolates were resistant and 15 of these were multi-drug resistant (MDR). Broth microdilution method showed that 68 isolates were resistant and 16 of these were MDR. Only two isolates, *B. brevis* B2-4 and *B. longum* B2-1, were susceptible to all 15 tested antimicrobials with both agar dilution method and broth microdilution method. Table 6 shows the total number of isolates of each species, the number of resistant isolates in each species to each antimicrobial, and the number of resistant isolates in each species to various antimicrobial. *L. plantarum* and *L. rhamnosus* were not only the most frequently used species in products (14 strains each) but also the most isolated species in this study. Kanamycin resistance was the most prevalent one (50 and 49 isolates with agar dilution method and broth dilution method, respectively), followed by chloramphenicol resistance (34 isolates with both methods). Number of resistant isolates to four antimicrobials (clindamycin, erythromycin, gentamicin, and streptomycin) assayed with the agar dilution method were larger than that assayed by the broth dilution method. On the contrary, numbers of resistant isolates to three antimicrobials (ampicillin, kanamycin, and tetracycline) assayed by the agar dilution method and the broth dilution method were the same. The agar dilution method and the broth dilution method produced different MICs for seven of nine antimicrobials (Table 7). Especially, differences in the number of resistant isolates were significant for clindamycin (17/25) and gentamicin (7/22). In addition, differences in MICs between the two methods were observed for different species: *L. acidophilus*, chloramphenicol-resistance and clindamycin-resistance; *L. fermentum*, chloramphenicol-resistance and clindamycin-resistance; *L. plantarum*, ampicillin-resistance and clindamycin-resistance and gentamicin-resistance. In case of *B. animalis* subsp. *lactis*, all six isolates were gentamicin-susceptible with the agar dilution method while all six isolates were gentamicin-resistant with the broth dilution method. Difference between the two methods was also observed for MDR type of each species (Table 7).

### Random Amplified Polymorphic DNA (RAPD)-PCR

According to 16S rRNA sequencing analysis, 70 isolates belonged to 17 species and twelve species had more than two isolates. Six isolates of *B. animalis* subsp. *lactis* showed the same band patterns. Ten isolates of *L. rhamnosus* showed different band patterns from each other while two isolates (*L. rhamnosus* 7-6 and *L. rhamnosus* 10) labelled as *L. rhamnosus* GG showed the same pattern as expected. For the remaining nine species, RAPD-PCR band patterns within the same species were different from one another (data shown in supplementary figures).

### Detection of Antimicrobial Resistant Genes

Only one *lnu*(A) gene and six *tet*(W) genes were detected with PCR using specific primers to 17 antimicrobial extrinsic resistant genes. One *lnu*(A) was detected in *L. reuteri* 16-1 which was resistant to ampicillin, lincomycin, and tetracycline but susceptible to clindamycin. Six *tet*(W) genes were detected in six *Bifidobacterium* isolates -three gentamicin- and tetracycline-resistant isolates (*B. animalis* subsp. *lactis* B3-2, B8-1, and B15-1) and three tetracycline-susceptible isolates (*B. longum* B1-1, B11, and B14-4).

### Conjugation

A total of eight isolates (two *Lactobacillus* isolates and six *Bifidobacterium*) were used as donors to test their ability to transfer tetracycline resistance to *E. faecalis* JH 2-2 and *Lc. lactis* subsp. *lactis* Bu2-60. No transconjugant could be obtained after filter mating of eight isolates tested.

## Discussion

Álvarez-Cisneros and Ponce-Alquicira [28] have demonstrated that resistance genes are not always expressed but can be transferred to other bacteria if environmental conditions stimulate the expression of these genes. An extrinsic resistance gene, whether it is expressed or not, can be transferred to microbiota. Many studies have reported that various LABs have different resistance genes that can be transferred to other bacteria. For instance, *erm*(A), *erm*(B), *tet*(M), *tet*(W), and *tet*(M) have been identified in several *Lactobacillus* species [17, 29]. Tetracycline resistance genes (*tet*(M), *tet*(S)) for ribosomal protection proteins and *tet*(L) for efflux pumps have been found in LABs [30]. Also, phenotypically susceptible strains containing genes for tetracycline (*tet*(K), *tet*(L)) and erythromycin (*erm*(B), *mef*(A)) resistance have been reported [31]. Ma *et al*. have shown that the presence of antimicrobial resistance gene does not always lead to expression of resistance [32].

The EFSA guideline [12] recommends that LAB for human consumption should be tested for their antimicrobial resistance. MFDS (Korea Ministry of Food and Drug Safety) guideline (2021) recommends confirmation of the absence of acquired or transferable antimicrobial resistance determinants by analyzing whole genome sequence.

In this study, MICs and PCR amplification of 17 antimicrobial-resistance extrinsic genes revealed discrepancies between the antimicrobial-resistance phenotype and actual detection of antimicrobial-resistant genes, similar to previous reports [28]. Although more than 95% of isolates were resistant to various antimicrobials, only two antimicrobial resistance genes (*lnu*(A) and *tet*(W)) were detected with PCR. Lincosamide nucleotidyl transferase gene (*lnu*(A)) was detected in one isolate of clindamycin-susceptible *L. reuteri* 16-1. Clindamycin susceptibility even in the presence of *lnu*(A) has been reported by others while *lnu*(A) is not detected with PCR in isolates with high MIC to clindamycin MIC [2. 11. 21, 32,] similar to what we observed in this study. Among nine tetracycline resistance genes, only *tet*(W) was detected in one isolate of tetracycline-susceptible *B. longum* (tetracycline MIC=2 μg/ml), two isolates of tetracycline-susceptible *B. animalis* subsp. *lactis* (MIC=8 μg/ml), and three tetracycline-resistant isolates of *B. animalis* subsp. *lactis* (tetracycline MIC=32 μg/ml). None of tet genes was detected in *L. reuteri* 16-1, *L. reuteri* 17-5, *L. rhamnosus* 2-7, or *L. fermentum*, although their tetracycline MICs were higher than 64 μg/ml. Other mechanism might be responsible for such high-level of tetracycline-resistance. Several LAB isolates had MICs higher than cut-off values to aminoglycoside group such as gentamicin, kanamycin, streptomycin, and neomycin (Table 5). However, the gene responsible for the aminoglycoside resistance (*aad*(E)) was not detected in these LABs as reported before by others [34].

As acquired resistance mediated by mobile genes may pose risk to the public health, it is important to determine whether the nature of resistance is intrinsic or acquired [2]. In general, AMR genes can be horizontally transferred from one microorganism to another by transduction or by transformation between microorganisms [28]. It has been reported that the primary mechanism to acquire resistance is by direct cell-to-cell contact or conjugation between different gene tra of bacteria, especially when resistant genes are present on mobile genetic elements such as plasmids and transposons [35].

In this study, several strains claimed on the labels were not isolated. Especially, two strains (*B. bifidum* and *L. delbrueckii* subsp. *lactis*) were not isolated from any products. It might be due to a low amount in the product or the loss of survivability. Only five isolates (by agar dilution method) or two isolates (by broth dilution method) were susceptible to all antimicrobials. Others are resistant to at least one antimicrobial and 20% of these resistant isolates were MDR. However, only two resistant genes were detected in both susceptible and resistant isolates, but not in MDR. This study suggests the importance to sequence the full genome to detect any extrinsic resistance gene that can be transferred to microbiota. In the past, antimicrobial resistance of LABs was considered as a good characteristic for LAB. Thus, resistant LABs were used as probiotics without knowledge of resistance problem. Our results suggest the need for continuous monitoring of newly registered probiotics as well as probiotics with a long history of use. In addition, the problem of MIC difference with agar dilution method and broth dilution method needs to be solved.

## Supplemental Materials

Supplementary data for this paper are available on-line only at http://jmb.or.kr.

## Figures and Tables

**Table 1:** Concentration ranges of antimicrobial susceptibility testing and acceptable ranges of quality control strains as suggested by ISO guideline.

Antimicrobial agent	Conc. range (μg/ml)	Quality control parameters			

		*Bifidobacterium longum* ATCC 15707	*Lacticaseibacillus paracasei* ATCC 334	*Lactiplantibacillus plantarum* ATCC 14917	*Lactococcus lactis* ATCC 19435
Ampicillin	0.032 to 16	0.25 to 1	0.5 to 2	0.25 to 2	0.12 to 1
Chloramphenicol	0.125 to 64	0.5 to 4	2 to 8	4 to 16	2 to 16
Clindamycin	0.032 to 16	0.03 to 0.12	0.06 to 0.25	0.5 to 4	0.25 to 1
Erythromycin	0.016 to 8	0.03 to 0.25	0.06 to 0.5	0.25 to 2	0.12 to 0.5
Gentamicin	0.5 to 256	4 to 32	1 to 4	─	0.5 to 2
Kanamycin	2 to 1024	64 to 512	16 to 64	─	2 to 8
Streptomycin	0.5 to 256	8 to 64	8 to 32	─	2 to 16
Tetracycline	0.125 to 64	0.5 to 2	1 to 4	8 to 32	0.5 to 2
Vancomycin	0.25 to 128	0.5 to 2	─	─	0.25 to 1

**Table 2:** Primers and PCR conditions for antimicrobial resistance genes tested in this study.

Resistance gene	Primers	Primer sequence (5′-> 3’)	*T*_a_(°C)	Amplicon size (bp)	Reference(s)
*aad*(E)	aadE-1	GCAGAACAGGATGAACGTATTCG	55	369	[17].
	aadE-2	ATCAGTCGGAACTATGTCCC			
*bla*Z	blaZ-1	CAGTTCACATGCCAAAGAG	52	846	[18].
	blaZ-2	TACACTCTTGGCGGTTTC			
cat	cat-1	TTAGGTTATTGGGATAAGTTA	44	300	[19].
	cat-2	GCATGRTAACCATCACAWAC			
*erm*(A)	ermA-1	AAGCGGTAAACCCCTCTGA	55	190	[20].
	ermA-2	TTCGCAAATCCCTTCTCAAC			
*erm*(B)	ermB-1	TTTTGAAAGCCGTGCGTCTG	55	202	[17]
	ermB-2	CTGTGGTATGGCGGGTAAGTT			
*erm*(C)	ermC-1	AATCGTCAATTCCTGCATGT	55	299	[20]
	ermC-2	TAATCGTGGAATACGGGTTTG			
*lnu*A (linA)	lnuA-1	GGTGGCTGGGGGGTAGATGTATTAACTGG	56	323	[21]
	lnuA-2	GCTTCTTTTGAAATACATGGTATTTTTCGATC			
*tet*(K)	tetK-1	CAATACCTACGATATCTA	50	352	[17]
	tetK-2	TTGAGCTGTCTTGGTTCA			
*tet*(L)	tetL-1	TGGTCCTATCTTCTACTCATTC	53	385	[22]
	tetL-2	TTCCGATTTCGGCAGTAC			
*tet*(M)	tetM-1	TCAACACATCGAGGTCCGTC	58	737	this study
	tetM-2	TCGCAACCATAGCGTATCCC			
*tet*(O)	tetO-1	AGCGTCAAAGGGGAATCACTATCC	55	1723	[17]
	tetO-2	CGGCGGGGTTGGCAAATA			
*tetB*(P)	TetB-1	AAAACTTATTATATTATAGTG	46	169	[23]
	TetB-2	TGGAGTATCAATAATATTCAC			
*tet*(Q)	TetQ-1	AGAATCTGCTGTTTGCCAGTG	63	169	[23]
	TetQ-2	CGGAGTGTCAATGATATTGCA			
*tet*(S)	tetS-1	ATCAAGATATTAAGGAC	55	573	[24. 25]
	tetS-2	TTCTCTATGTGGTAATC′			
*tet*(T)	TetT-1	AAGGTTTATTATATAAAAGTG	46	169	[23]
	TetT-2	AGGTGTATCTATGATATTTAC			
*tet*(W)	tetW-1	ATATTGGAATTCTTGCCCAT	48	510	this study
	tetW-2	ATGCTTCTATGTCGGTATTT			
*tet*(M) group	tetMgr-1	GAYACICCIGGICAYRTIGAYTT	45	1100	[26].
	tetMgr-2	GCCCARWAIGGRTTIGGIGGIACYTC			

**Table 3:** Primers for random amplified polymorphic DNA analysis.

No.	Primer name	Primer sequence (5’->3’)
1	RP1	GGT GAG GGA A
2	RP2	GTT TCG CTC C
3	RP3	GTA GAC CCG T
4	RP4	AAG AGC CCG T
5	RP5	AAC GCG CAA C
6	RP6	CCC GTC AGC A
7	RP7	GAA ACG GGT G
8	RP8	TCG GCG ATA G
9	RP9	ACG CGC CCT
10	RP10	GTT TTC CCA GTC ACG AC

**Table 4:** Number of strains in 21 top-selling probiotic products studied in this study.

Sample No.	Number of total strains claimed by the product^a^	Number of strains belonged to *Bifidobacterium*, *Lactobacillus*, and *Lactococcus*	

		Claimed by the product^b^	Isolated from the product^c^
S1	6	5	4
S2	25	22	6
S3	6	4	3
S4	10	9	3
S5	4	3	1
S6	2	2	2
S7	9	8	4
S8	12	10	6
S9	1	1	1
S10	1	1	1
S11	2	2	2
S12	1	1	1
S13	1	1	1
S14	12	10	7
S15	1	1	1
S16	2	2	2
S17	19	16	10
S18	18	16	7
S19	7	6	3
S20	11	9	3
S21	2	2	2
Total	152	131	70

^a^Total number of strains claimed on the product belonged to the genus *Bifidobacterium*, *Bacillus*, *Enterococcus*, *Lacticaseibacillus*, *Lactiplantibacillus*, *Lactobacillus*, *Lactococcus*, *Latilactobacillus*, *Levilactobacillus*, *Ligilactobacillus*, *Limosilactobacillus*, and *Streptococcus*; ^b^Number of strains belonged to the genus *Bifidobacterium* *Lacticaseibacillus*, *Lactiplantibacillus*, *Lactobacillus*, *Lactococcus*, *Latilactobacillus*, *Levilactobacillus*, *Ligilactobacillus*, *Limosilactobacillus* species claimed on the label of the product; ^c^Number of isolates belonged to the genus *Bifidobacterium*, *Lacticaseibacillus*, *Lactiplantibacillus*, *Lactobacillus*, *Lactococcus*, *Latilactobacillus*, *Levilactobacillus*, *Ligilactobacillus*, *Limosilactobacillus* from each product.

**Table 5:** Minimum inhibitory concentrations (MICs) of 9 antimicrobials by agar and broth-micro dilution methods to 70 LABs isolated from 21 commercial products.

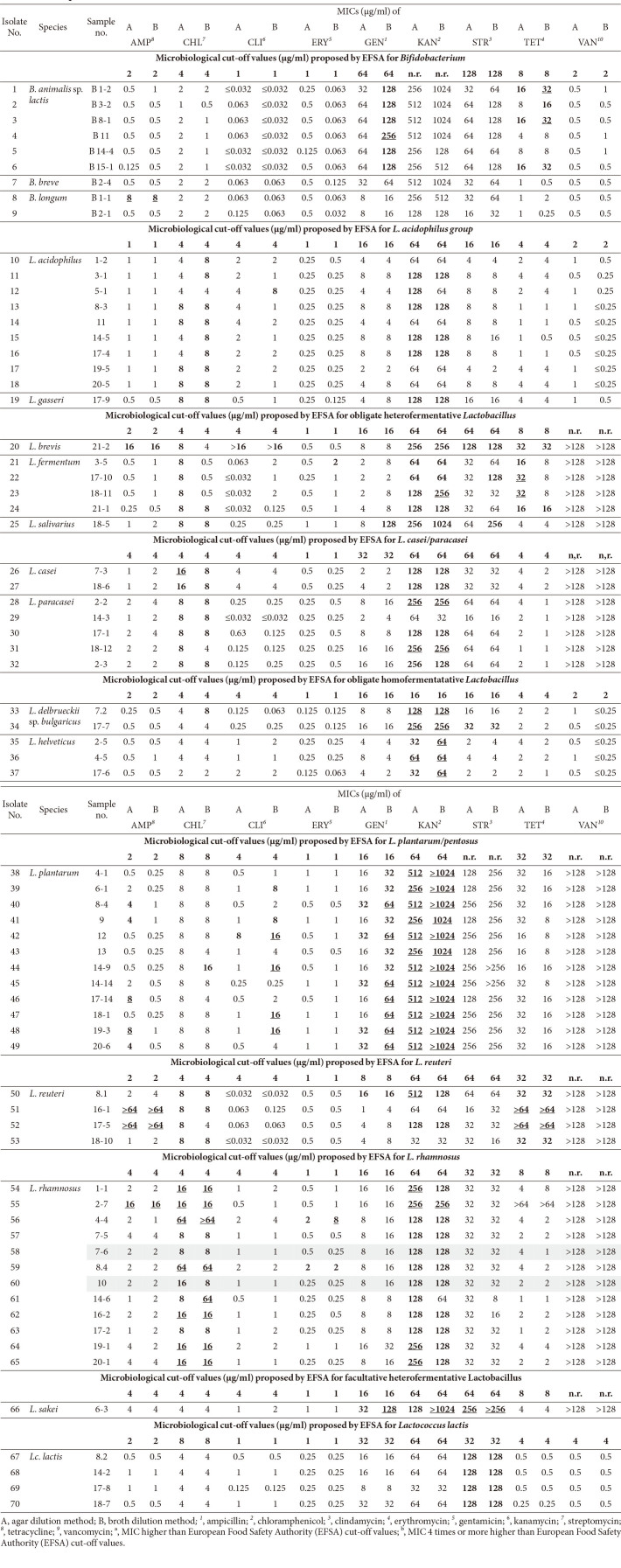

**Table 6:** Number of antimicrobial resistant isolates to each antimicrobial.

Species	No. of isolates/ Total no. of strains claimed on the product^a^	AMP		CHL		CLI		ERY		GEN		KAN		STR		TET		VAN		No. of resistant isolates^b^ (%)		No. of strong resistant isolates^c^ (%)		No. of MDR isolates^d^	
		A	B	A	B	A	B	A	B	A	B	A	B	A	B	A	B	A	B	A	B	A	B	A	B
*Bifidobacterium animalis* sp *lactis*	6/12	0	0	0	0	0	0	0	0	0	6	0	0	0	0	3	4	0	0	3	6	0	4	0	0
*Bifidobacterium breve*	1/9	0	0	0	0	0	0	0	0	0	0	0	0	0	0	0	0	0	0	0	0	0	0	0	0
*Bifidobacterium longum*	2/10	1	1	0	0	0	0	0	0	0	0	0	0	0	0	0	0	0	0	1	1	1	1	0	0
*Lactobacillus. acidophilus*	9/13	0	0	4	8	0	1	0	0	0	0	5	4	0	0	0	0	0	0	8	9	0	0	0	0
*Levilactobacillus brevis*	1/1	1	1	1	0	1	1	0	0	0	0	1	1	1	1	1	1	0	0	1	1	1	1	1	1
*Lacticaseibacillus casei*	3/7	0	0	2	2	0	0	0	0	0	0	2	2	0	0	0	0	0	0	2	2	2	0	0	0
*Lactobacillus delbrueckii* sp. *bulgaricus*	1/4	0	0	0	1	0	0	0	0	0	0	2	2	1	1	0	0	0	0	2	2	2	2	0	0
*Limosilactobacillus fermentum*	4/6	0	0	4	1	0	0	0	1	0	0	2	2	0	1	4	1	0	0	4	4	2	1	2	1
*Lactobacillus gasseri*	1/3	0	0	1	1	0	0	0	0	0	0	1	1	0	0	0	0	0	0	1	1	0	0	0	0
*Lactobacillus helveticus*	3/5	0	0	0	0	0	0	0	0	0	0	3	3	0	0	0	0	0	0	3	3	1	3	0	0
*Lacticaseibacillus paracasei*	4/6	0	0	5	4	0	0	0	0	0	0	4	4	0	0	0	0	0	0	5	5	3	2	0	0
*Lactiplantibacillus plantarum*	12/14	5	0	0	1	1	6	0	0	5	12	12	12	0	0	0	0	0	0	12	12	12	12	0	1
*Limosilactobacillus reuteri*	4/5	2	3	4	3	0	0	0	0	1	1	2	2	0	0	2	2	0	0	4	4	3	2	2	3
*Lacticaseibacillus rhamnosus*	12/14	1	1	12	12	0	0	2	2	0	1	12	11	0	0	1	1	0	0	12	12	8	8	3	3
*Latilactobacillus sakei*	1/1	0	0	0	0	0	0	0	0	1	1	1	1	1	1	0	0	0	0	1	1	1	1	0	0
*Ligilactobacillus salivarius*	1/3	0	0	1	1	0	0	0	0	0	1	1	1	0	1	0	0	0	0	1	1	1	1	0	0
*Lactococcus lactis*	4/6	0	0	0	0	0	0	0	0	0	0	0	0	4	4	0	0	0	0	4	4	4	4	0	0
*Total*	70/119	10	6	34	34	2	8	2	3	7	22	48	46	7	9	11	9	0	0	64 (91.4%)	68 (97.1%)	41 (58.6%)	42 (60%)	8 (11.4%)	9 (12.9%)

A, agar dilution method; B, broth microdilution method; ^a^Eleven strains of *B. bifidum* and one strain of *L. delbrueckii* subsp. *lactis* claimed on the labels of products were not recovered, so they were excluded from the Table. ^b^number of isolates with MIC higher than the cut-off value; ^c^number of isolates with MIC more than 4 times of the cut-off value; ^d^number of isolates which are resistant to more than three antimicrobials

**Table 7:** Multi-drug resistance types of each antimicrobial resistant isolate.

MDR isolate	Type of MDR	

	A	B
*L. brevis* 21-2	AMP^*r*^ **CHL^*r*^** CLI^*r*^ KAN^*r*^ STR^*r*^ TET^*r*^	AMP^*r*^ CLI^*r*^ KAN^*r*^ STR^*r*^ TET^*r*^
*L. fermentum* 18-11	**CHL^*r*^** KAN^*r*^ **TET^*r*^**	KAN^*r*^
*L. fermentum* 21-1	CHL^*r*^ KAN^*r*^ TET^*r*^	CHL^*r*^ KAN^*r*^ TET^*r*^
*L. plantarum* 14-9	KAN^*r*^	**CHL^*r*^ CLI^*r*^ GEN^*r*^** KAN^*r*^
*L. reuteri* 8-5	CHL^*r*^ GEN^*r*^ KAN^*r*^	**AMP^*r*^** CHL^*r*^ GEN^*r*^ KAN^*r*^
*L. reuteri* 16-1	AMP^*r*^ CHL^*r*^ TET^*r*^	AMP^*r*^ CHL^*r*^ TET^*r*^
*L. reuteri* 17-5	AMP^*r*^ **CHL^*r*^** KAN^*r*^ TET^*r*^	AMP^*r*^ KAN^*r*^ TET^*r*^
*L. rhamnosus* 2-7	AMP^*r*^ CHL^*r*^ KAN^*r*^ TET^*r*^	AMP^*r*^ CHL^*r*^ KAN^*r*^ TET^*r*^
*L. rhamnosus* 4-4	CHL^*r*^ ERY^*r*^ KAN^*r*^	CHL^*r*^ ERY^*r*^ KAN^*r*^
*L. rhamnosus* 8-7	CHL^*r*^ ERY^*r*^ KAN^*r*^	CHL^*r*^ ERY^*r*^ KAN^*r*^

A, agar dilution method; B, broth microdilution method; Boldface indicates antimicrobial showing resistance only one of two methods.

**Table 8:** Number of resistant isolates belonged to each species.

Species	Number of isolates	Number of isolates resistant to each antimicrobial
*B. animalis*	6	GEN (0/6) TET (3/4)
*B. breve*	1	—
*B. longum*	2	AMP (1/1)
*L. acidophilus*	9	CHL (4/8) CLI (0/1) KAN (5/4)
*L. brevis*	1	AMP (1/1) CHL (1/0) CLI (1/1) KAN (1/1) STR (1/1) TET (1/1)
*L. casei*	2	CHL (2/2) KAN (2/2)
*L. delbrueckii*	2	CHL (0/1) KAN (2/2) STR (1/1)
*L. fermentum*	4	CHL (4/1) ERY (0/1) KAN (2/2) STR (0/1) TET (4/1)
*L. gasseri*	1	CHL (1/1) KAN (1/1)
*L. helveticus*	3	KAN (3/3)
*L. paracasei*	5	CHL (5/4) KAN (4/4)
*L. plantarum*	12	AMP (5/0) CHL (0/1) CLI (1/6) GEN (5/12) KAN (12/12)
*L. reuteri*	4	AMP (2/3) CHL (4/3) GEN (1/1) KAN (2/2) TET (2/2)
*L. rhamnosus*	12	AMP (1/1) CHL (12/12) ERY (2/2) GEN (0/1) KAN (12/11) TET (1/1)
*L. sakei*	1	GEN (1/1) KAN (1/1) STR (1/1)
*L. salivarius*	1	CHL (1/1) GEN (0/1) KAN (1/1) STR (0/1)
*Lc. lactis*	4	STR (4/4)
Number of resistant isolates	70	AMP (10/6) CHL (34/34) CLI (2/8) ERY (2/3) GEN (7/22) KAN (48/46) STR (7/9) TET (11/9) VAN (0/0)
