# Supplementary material for: Antimicrobial Resistance of Seventy Lactic Acid Bacteria Isolated from Commercial Probiotics in Korea
Source: J Microbiol Biotechnol. 2023 Jan 12;33(4):500–10. doi: 10.4014/jmb.2210.10041 (PMC10164723; doi:10.4014/jmb.2210.10041)
Supplement: Supplementary file 1 [file jmb-33-4-500-supple.pdf]

J. Microbiol. Biotechnol.
https://doi.org/10.4014/jmb.2210.10041

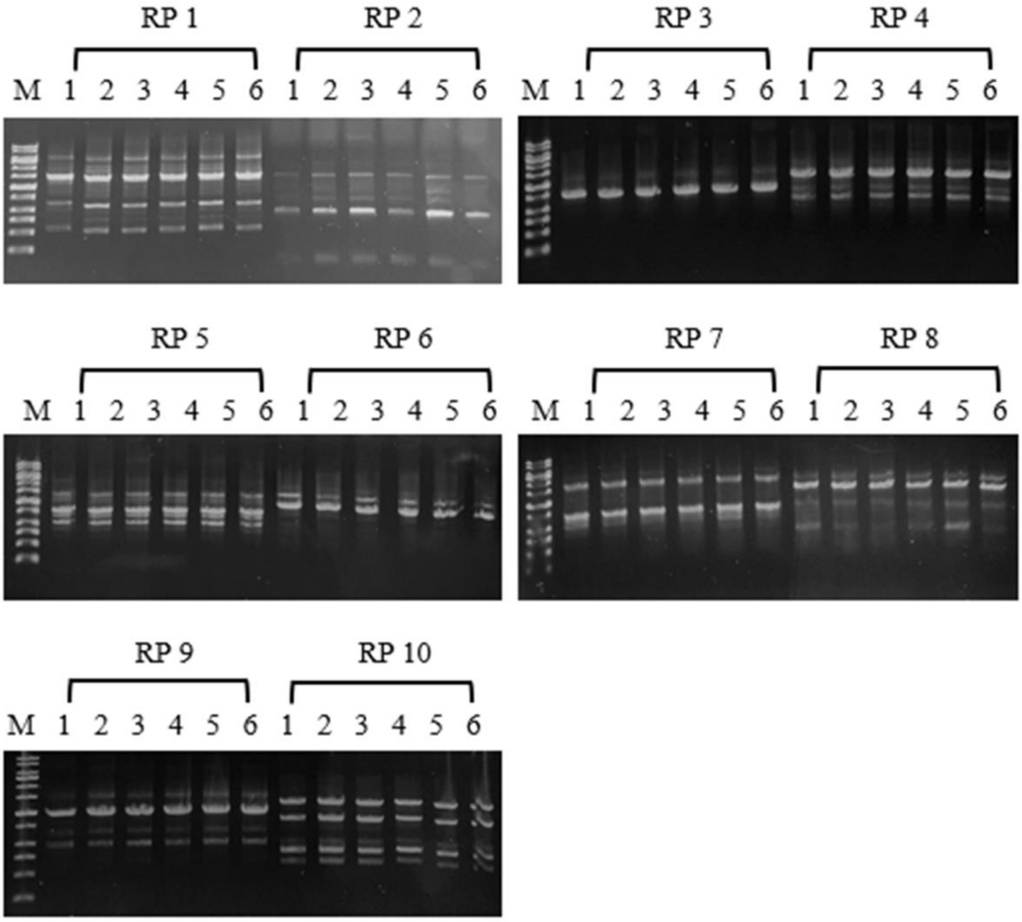

Fig. S1. RAPD-PCR analysis was performed using ten selected primers (Table 2). RAPD-PCR profiles obtained from *Bifidobacterium animalis* subsp._*lactis* isolates electrophoresed on 1% TAE agarose gel. M, 1 kb DNA size marker; 1, B1-2; 2, B3-2; 3, B8-1; 4, B11; 5, B14-4; 6, B15-1. In all cases, the electrophoretic patterns are identical for the same primer.

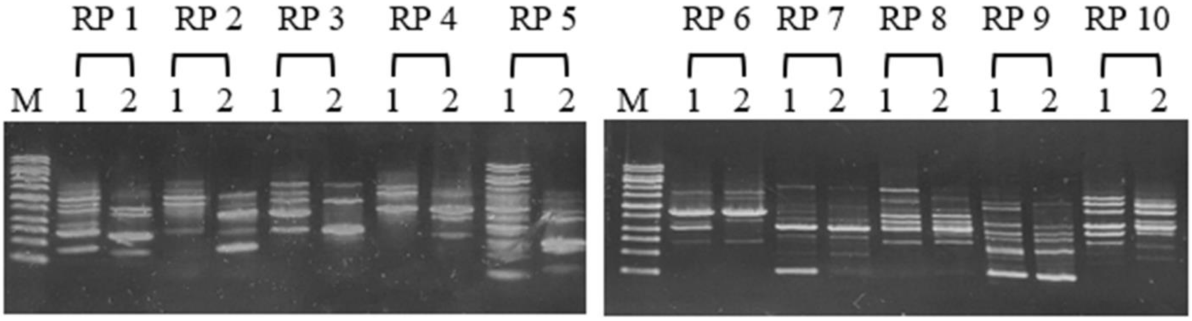

Fig. S2. RAPD-PCR analysis was performed using ten selected primers (Table 2). RAPD-PCR profiles obtained from *Bifidobacterium longum* isolates electrophoresed on 1% TAE agarose gel. M, 1 kb DNA size marker; 1, B1-1; 2, B2-1

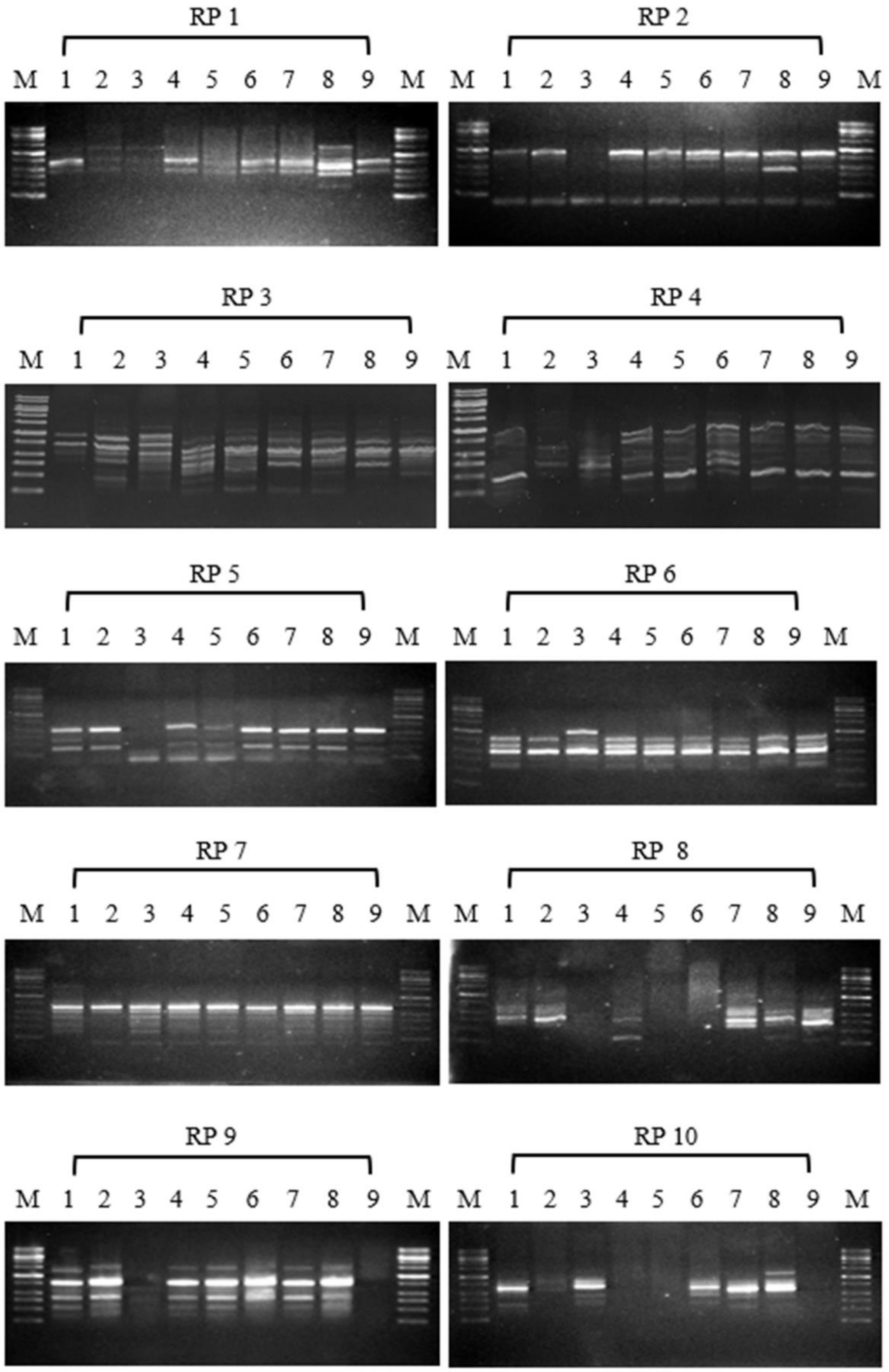

Fig. S3. RAPD-PCR analysis was performed using ten selected primers (Table 2). RAPD-PCR profiles obtained from *Lactobacillus acidophilus* isolates electrophoresed on 1% TAE agarose gel. M, 1 kb DNA size marker; 1, 1-2; 2, 3-1; 3, 5-1; 4, 8-3; 5, 11; 6, 14-5; 7, 17-4; 8, 19-5; 9, 20-5

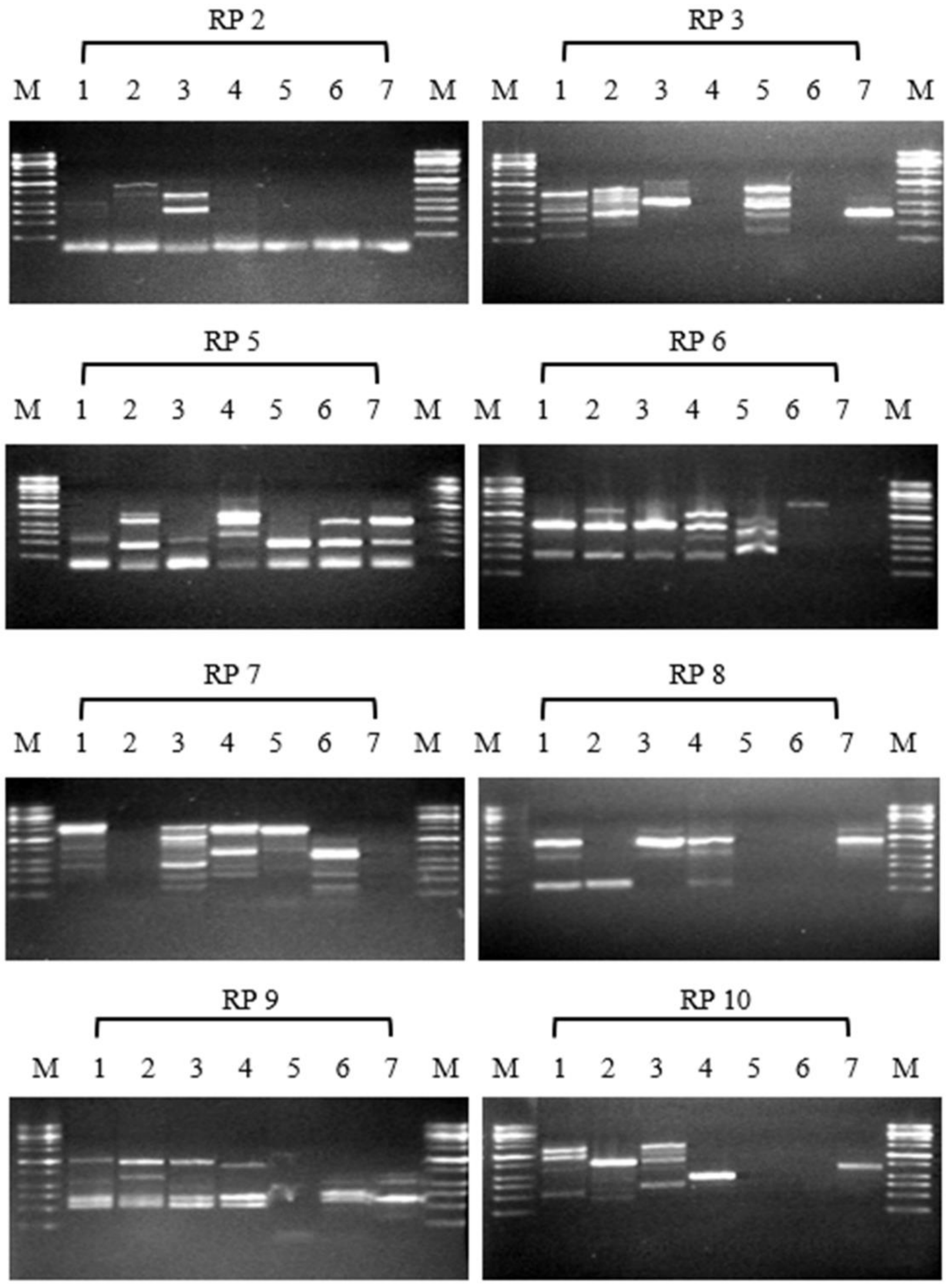

Fig. S4. RAPD-PCR analysis was performed using eight selected primers (Table 2). RAPD-PCR profiles obtained from *Lacticaseibacillus paracasei and casei isolates* electrophoreses on 1% TAE agarose gel. M, 1 kb DNA size marker; 1, 2-2; 2, 14-3; 3,17-1; 4, 18-12; 5, 2-3; 6, 7-3; 7, 18-6

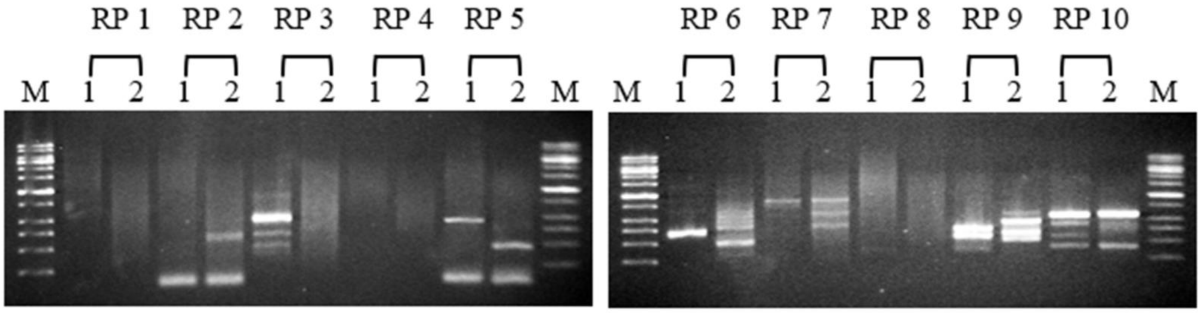

Fig. S5. RAPD-PCR analysis was performed using ten selected primers (Table 2). RAPD-PCR profiles obtained from *Lactobacillus delbrueckii* subsp. *bulgaricus* isolates electrophoresed on 1% TAE agarose gel. M, 1 kb DNA size marker; 1, 7-7; 2, 17-7

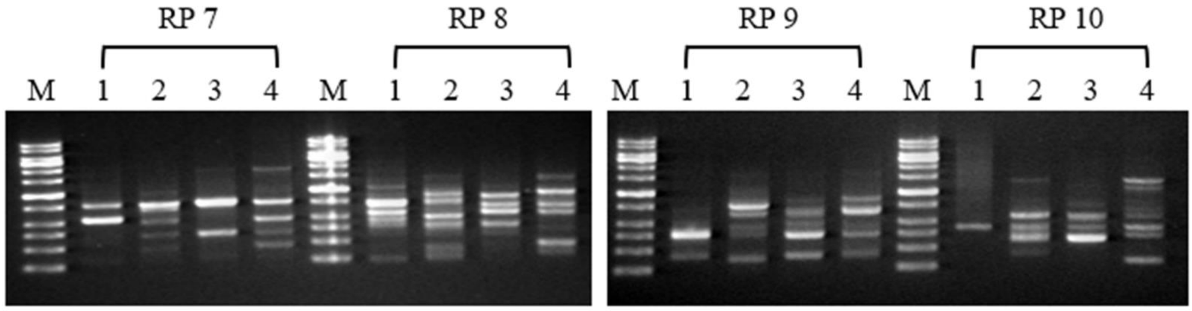

Fig. S6. RAPD-PCR analysis was performed using four selected primers (Table 2). RAPD-PCR profiles obtained from *Limosilactobacillus fermentum* isolates electrophoresed on 1% TAE agarose gel. M, DNA size marker; 1, 3-5; 2, 17-10; 3, 18-11, 4, 21-1

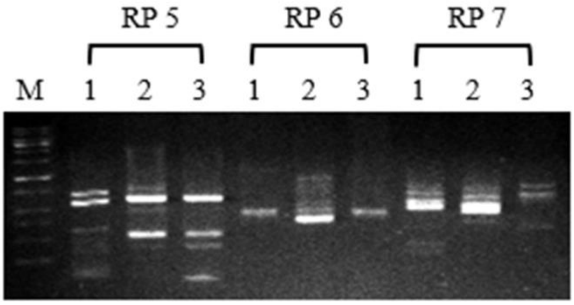

Fig. S7. RAPD-PCR analysis was performed using three selected primers (Table 2). RAPD-PCR profiles obtained from *Lactobacillus helveticus* isolates electrophoresed on 1% TAE agarose gel. M, 1 kb DNA size marker; 1, 2-5; 2, 4-5; 3, 17-6

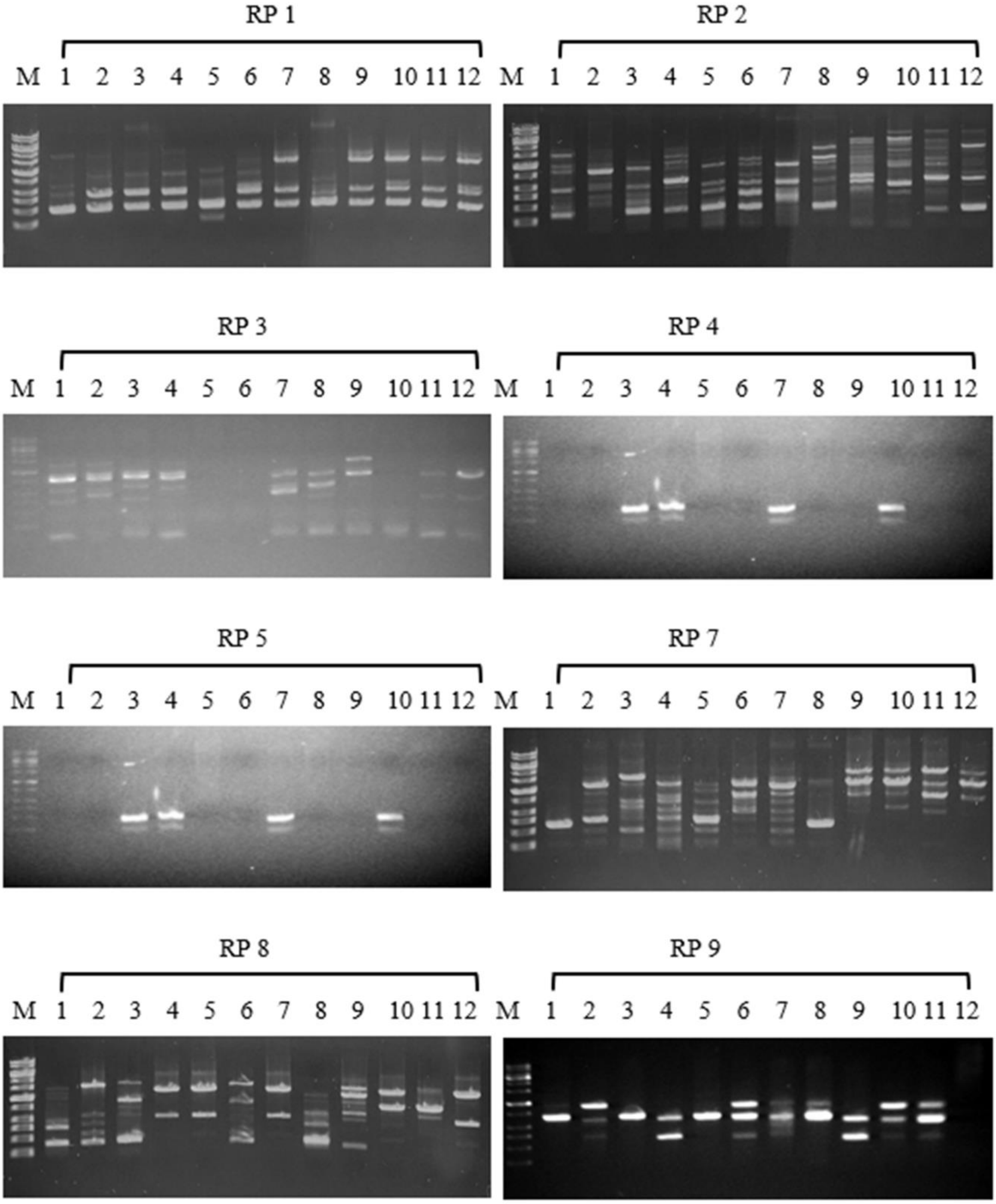

Fig. S8. RAPD-PCR analysis was performed using eight selected primers (Table 2). RAPD-PCR profiles obtained from *Lactiplantibacillus plantarum* isolates electrophoresed on 1% TAE agarose gel. M, DNA size marker; 1, 4-1; 2, 6-1; 3, 8-4; 4, 9; 5, 12; 6, 13; 7, 14-9; 8, 14-14; 9, 17-14; 10, 18-1; 11, 19-3; 12, 20-6

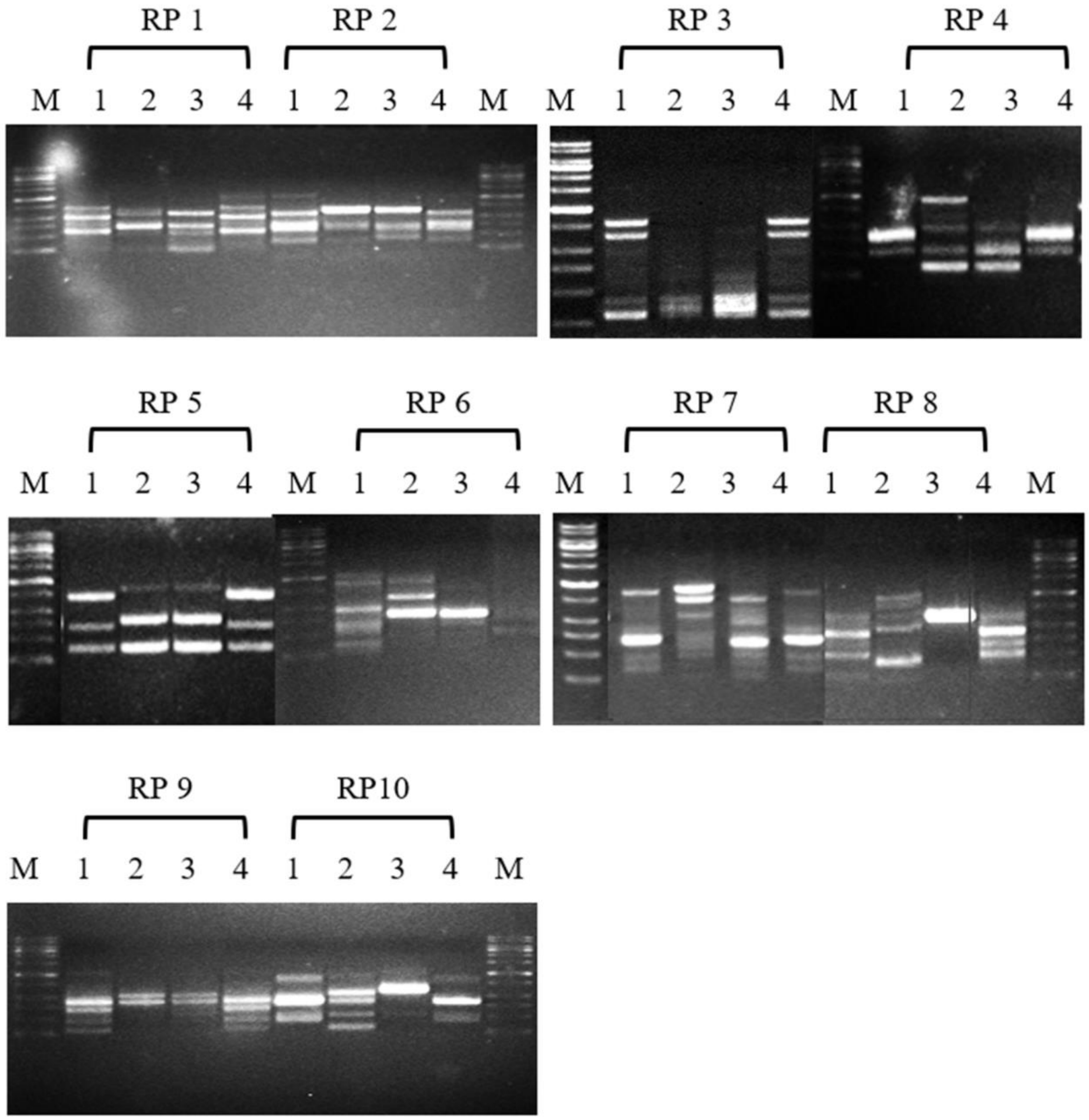

Fig. S9. RAPD-PCR analysis was performed using ten selected primers (Table 2). RAPD-PCR profiles obtained from *Limosilactobacillus reuteri* isolates electrophoresed on 1% TAE agarose gel. M, 1 kb DNA size marker; 1, 8-5; 2, 16-1; 3, 17-5; 4, 18-10

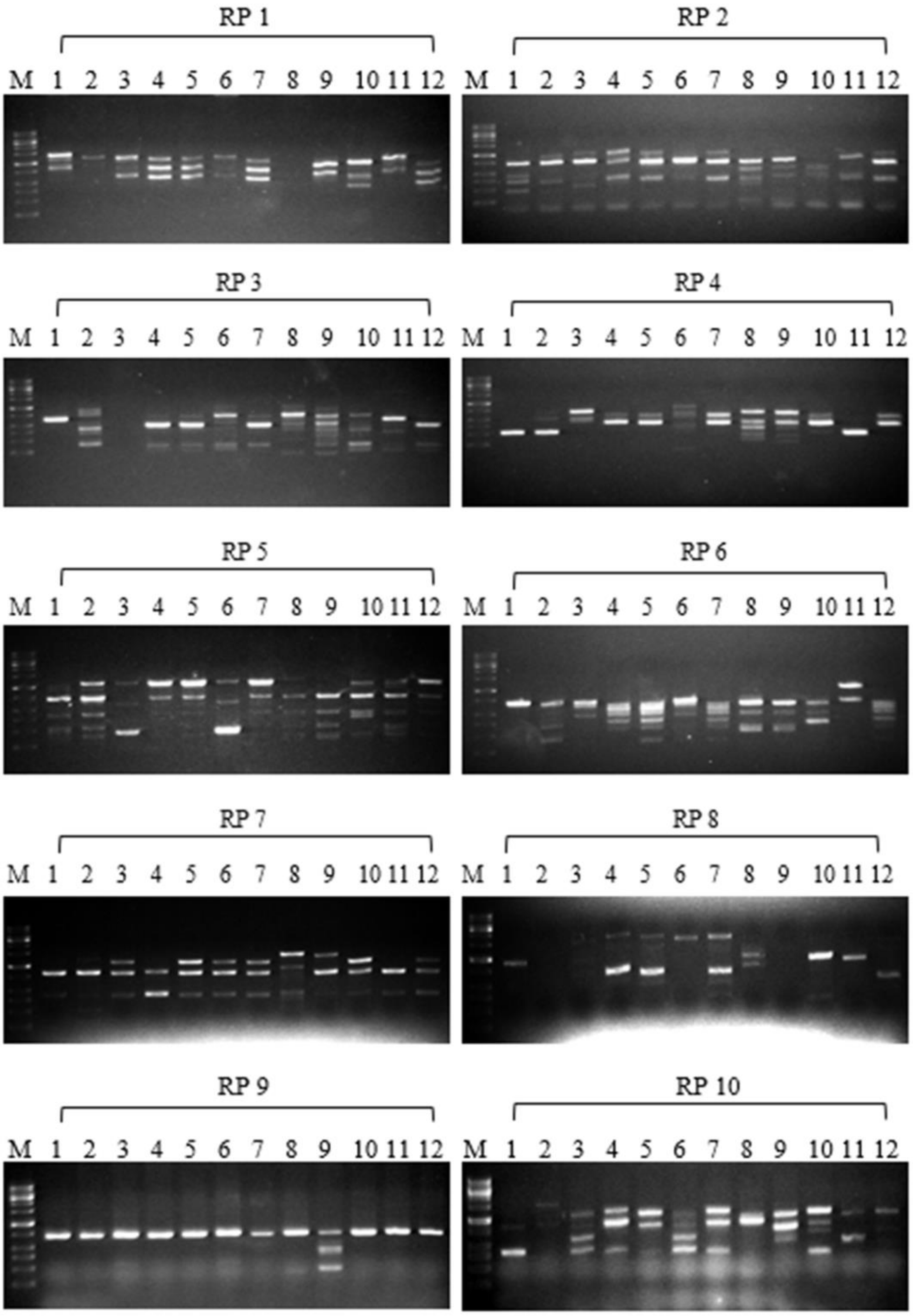

Fig. S10. RAPD-PCR profiles obtained from *Lacticaseibacillus rhamnosus* isolates electrophoresed on 1% TAE agarose gel. M, 1 kb DNA size marker; 1, 1-1; 2, 2-7; 3, 4-4; 4, 7-5; 5, 7-6; 6, 8-7; 7, 10; 8, 14-6; 9, 16-2; 10, 17-2; 11, 19-1; 12, 20-1. *Lb. rhamnosus* 7-6 and 10 isolates produced identical electrophoretic patterns for 10 selected primers.

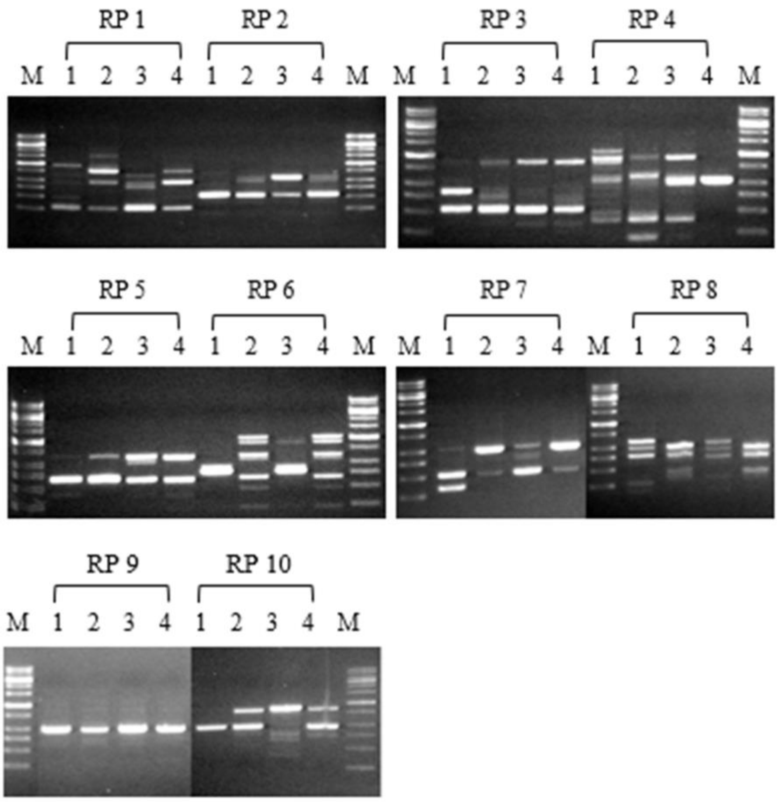

Fig. S11. RAPD-PCR analysis was performed using ten selected primers (Table 2). RAPD-PCR profiles obtained from *Lactococcus lactis* isolates electrophoresed on 1% TAE agarose gel. M, kb DNA size marker; 1, 8-6; 2, 14-2; 3, 17-8; 4, 18-7
